# Clinical variation in Lowe syndrome: what and how?

**DOI:** 10.3389/fcell.2025.1720452

**Published:** 2025-11-06

**Authors:** Eileen D. Brewer

**Affiliations:** Division of Pediatric Nephrology, Department of Pediatrics, Baylor College of Medicine, Houston, TX, United States

**Keywords:** Lowe syndrome, oculocerobrorenal, ocrl, phenotype, genotype, dent disease 2, kidney, neurologic

## Abstract

Lowe syndrome is an X-linked disorder caused by mutations of the *OCRL* gene which encodes the enzyme inositol polyphosphate-5-phosphatase OCRL (Ocrl1) and is expressed in almost all body cells. Clinical characteristics involve kidney, brain, eye, muscle, bone, teeth, testes, skin and thrombocytes. Clinical phenotypes are heterogenous among families and even among affected boys in the same family. All have kidney disease varying from severe manifestations of Fanconi syndrome to only low molecular weight proteinuria, hypercalciuria and little kidney disease in the first decade of life. All develop chronic kidney disease (CKD) that typically progresses slowly and reaches stages 4–5 after the second or third decade. All have neurological dysfunction, including developmental delay, marked intellectual impairment and behavioral abnormalities; ∼50% have seizure disorder. Congenital cataracts with or without glaucoma are almost always present. Less common features are hypotonia, bone abnormalities unrelated to kidney disease, abnormal teeth, cryptorchidism, skin cysts and mild bleeding disorder. Although Lowe syndrome is a monogenic disease, genotype/phenotype correlation is difficult to establish. Ubiquitous expression and complexity of Ocrl1 function likely contribute to the elusiveness of correlation. Additionally, two diseases, Lowe syndrome and Dent disease type 2, result from mutations in the *OCRL* gene with some overlap in affected exons. Growing research in molecular and conformational abnormalities of Ocrl1 variants is triggering development of cell phenotype models for further study. Understanding how genotype leads to clinical phenotype has potential to provide better predictors of Lowe syndrome severity and specific therapeutic strategies for different subsets of affected patients.

## Introduction

Lowe syndrome (oculo-cerebro-renal disease of Lowe) is a rare X-linked disorder with no specific therapy caused by pathologic variants of the *OCRL* gene (Lewis et al., 2019; Bokenkamp and Ludwig, 2016). Over 200 mutations are associated with Lowe syndrome, most being missense/nonsense variants (49%), but also splicing (11%), small deletion (20%), small insertion (10%) or gross deletion (6%) variants (HGMD, 2021). The *OCRL* gene encodes for inositol polyphosphate-5-phosphatase OCRL (Ocrl1), a 901 amino-acid containing protein which catalyzes hydrolysis of the 5-position phosphate of the inositol ring of phosphoinositide lipids, the most abundant of which is phosphatidylinositol 4,5-bisphosphate. Ocrl1 occurs in cells of most body tissues and in multiple subcellular locations including the Golgi network, clathrin-coated vesicles, early endosomes and the plasma membrane (NCBI, 2025). Through both its enzyme activity and its many protein-protein interactions, Ocrl1 plays multiple roles in cell protein signaling and trafficking for endocytosis and phagocytosis, for metabolism in stressed lysozymes, for actin cytoskeleton remodeling and cell shape adjustments, for Golgi apparatus fragmentation and for ciliogenesis, (De Matteis et al., 2017; Ramadesikan et al., 2021; De Sa et al., 2025). As might be expected from the widespread distribution and complexity of Ocrl1 function, clinical characteristics of Lowe syndrome may involve many organs including the classic triad of kidney, brain and eye as well as muscle, bone, teeth, testes, skin and blood thrombocytes (Lewis et al., 2019). Different gene variants likely lead to different clinical phenotypes, but correlation of genotype with phenotype has proven to be complex (Zaniew et al., 2018; Hichri et al., 2011; Zhang et al., 2022; Du et al., 2024; Ando et al., 2024; Akhtar et al., 2022). Growing research interest in the molecular and conformational abnormalities of Ocrl1 does show correlation between gene variants and pathogenic cellular Ocrl1 function and location (Ramadesikan et al., 2021; De Sa et al., 2025; Lee J. J. et al., 2023). Understanding genotype to phenotype correlation may lead in the future to prediction of disease severity and specific therapeutic strategies for different subsets of affected patients. This paper will review the current clinical and cellular phenotype variations of Lowe syndrome, what is known about their correlation with *OCRL* genotypes and where future research is headed.

## Clinical Phenotype Variation

Clinical phenotypes of Lowe syndrome are heterogenous, varying among patients and even among affected males in the same family (Zaniew et al., 2018; Zhang et al., 2022; Du et al., 2024; Ando et al., 2024; Akhtar et al., 2022). Dent disease type 2 (Dent 2), which also results from mutations in the *OCRL* gene, has similar clinical phenotypes, but they are milder and predominantly involve the kidney (Table 1). Lowe syndrome occurs in all ethnicities worldwide. All Lowe syndrome patients have kidney and neurologic manifestations and about 90% have short stature that is not related to severity of kidney disease (Lewis et al., 2019; Bokenkamp and Ludwig, 2016). Kidney problems are mostly related to proximal tubular dysfunction and vary in severity from partial or full blown Fanconi syndrome (low molecular weight proteinuria (LMWP), aminoaciduria, bicarbonaturia with renal tubular acidosis, phosphaturia with hypophosphatemia and rickets, glucosuria, sodium and potassium wasting and polyuria) to only LMWP, hypercalciuria with kidney calcinosis/stones or little kidney disease at all in the first decade of life. Glucosuria is uncommon. LMWP is present from birth. Other features of the Fanconi syndrome do not manifest until after the first few months of life. Slowly progressive chronic kidney disease (CKD) eventually occurs in all patients but usually does not reach stages 2–5 until after 10 years of age (Zaniew et al., 2018; Ando et al., 2024). CKD likely results from progressive proximal tubular injury with subsequent tubulointerstitial fibrosis and likely nephron death, although the exact pathogenesis is not entirely clear (Lewis et al., 2019; Bokenkamp and Ludwig, 2016). Progression to end-stage kidney disease is typically delayed until the third or fourth decade of life (Ando et al., 2024). All Lowe syndrome patients have some neurological dysfunction with variable degrees of developmental delay, intellectual impairment (mean IQ 40-50) and abnormal behaviors, typically stubbornness, temper tantrums, repetitive purposeless movements like hand biting/flapping (stereotypies) and stranger anxiety. Seizures occur in up to 50% (Bokenkamp and Ludwig, 2016). Almost all Lowe syndrome patients have congenital cataracts and about 50% also have glaucoma (Song et al., 2017; Ma et al., 2020). The Table shows the rare absence of cataracts (missing in a single 6-year-old patient) in a small series of 8 unrelated southern Chinese patients with known *OCRL* gene variants (Du et al., 2024). These patients were young with 7 patients 1-6 years old and one patient 13 years old.

**TABLE 1:** Frequency of prominent clinical features.

Clinical Feature	%Lowe syndrome patients			%Dent 2 patients
	2024 Japan*	2024 China**	2018 Europe/Asia***	2018 Europe/Asia***
Number of patients	54	8	88	18
Age range (years)	2–45	1–13	1–18.5	1–18.5
LMWP	100%	100%	100%	100%
Short stature	89	100	91	50
Aminoaciduria	n/a	100	78	22
Phosphaturia/rickets	43	38	57	10
Bicarbonaturia/RTA	74	38	59	6
Glucosuria	n/a	25	15	22
Complete FS	n/a	n/a	7	0
Hypercalciuria	77	38	83	67
Kidney calcinosis/stones	35	25	52	50
Kidney stones	n/a	n/a	22	33
CKD, stage 2–3	69	n/a	73	61
CKD, stage 4–5	31	n/a	11	0
Hypotonia/high CK/LDH/AST	n/a	88		
Cataracts (congenital)	100	88		
Cognitive/behavioral issues	100	100		
Seizures	n/a	25		

Data adapted from *Ando et al. (2024), **Du et al. (2024) and ***Zaniew et al. (2018). Zaniew et al. only focused on kidney disease, so reported no data for extra-kidney manifestations. Gianesello et al. (2021) reviewed all published data for Dent 2 through 2020 and reported frequency of growth failure 64% (n = 28) and extra-kidney symptoms in muscle (elevated serum CK/LDH) 68% (n = 59), eye (mild cataracts, not congenital) 11% (n = 104) and nervous system (mild cognitive/behavioral issues) 34% (n = 97); 41% (n = 95) had no extra-kidney symptoms, 35% had symptoms in only 1 organ system; only 4% had symptoms in all three—muscle, eye and nervous system. LMWP, low molecular weight proteinuria; RTA, renal tubular acidosis; FS, fanconi syndrome; CKD; chronic kidney disease; CK, creatine kinase; LDH, lactate dehydrogenase; AST, aspartate aminotransferase; n/a, not available in dataset.

The classic clinical features of Lowe syndrome for kidney, brain and eye vary in frequency of occurrence (Table 1). In a large international cohort of 88 affected boys <19 years of age (Zaniew et al., 2018), analysis for kidney disease showed the most common features were the result of proximal tubular dysfunction, including LMWP (100%), aminoaciduria (78%), hypercalciuria (83%), phosphaturia (57%) and bicarbonaturia/renal tubular acidosis (59%), all features of Fanconi syndrome. Glucosuria was uncommon (15%), and most patients only had partial Fanconi syndrome with just 7% exhibiting complete Fanconi syndrome.

LMWP is universally present in patients with Lowe syndrome and is apparent from birth (Lewis et al., 2019). LMW proteins (<60 kDa), including retinol-binding protein, beta-2-microglobulin and the lysosomal enzyme N-acetylglucosaminidase, are normally filtered by the glomerulus and completely reabsorbed in the proximal tubule through clathrin-mediated and megalin-cubilin receptor endocytosis, then metabolized in lysosomes (Lewis et al., 2019; De Matteis et al., 2017). Ocrl1 is pivotal in regulating at least a portion of this process (De Matteis et al., 2017) and disruption in Lowe syndrome leads invariably to LMWP. Albuminuria, which is widely regarded as a marker for glomerular injury (Makhammajanov et al., 2024), also occurs in Lowe syndrome but is likely indicative of proximal tubular dysfunction and not related to glomerular disease. Ocrl1 is present in glomerular endothelial cells, mesangial cells and podocytes and may play a role in maintenance of the podocyte protein filtration barrier (Preston et al., 2020). More studies are needed to confirm whether Ocrl1 variants have any role in Lowe syndrome glomerular pathology. Whether abnormal glomerular filtration may contribute to albuminuria in late stages of CKD in Lowe syndrome is unknown, but increased albumin filtration is unlikely to cause significant further injury to an already dysfunctional proximal tubule. Albumin (69 kDa) is at the upper limit of size for proteins normally filtered by the glomerulus. Small amounts of albumin (estimated 3-5 g) are filtered daily by normal glomeruli and almost completely (80%) reabsorbed in normal proximal tubules by some of the same endocytosis processes as for LMW proteins (Tojo and Kinugasa, 2012; Molitaris et al., 2022). The other 20% of albumin reabsorption occurs in the loop of Henle and distal tubule, leaving the final urine with scarcely any to no albumin. All Lowe syndrome patients have albuminuria/proteinuria and more than half have proteinuria in the nephrotic range >1 g/m2/day; however, serum albumin remains normal to mildly elevated and is never low (Lewis et al., 2019; Zaniew et al., 2018). In diseases like diabetes and glomerulonephritis, excess filtration of albumin and other proteins by diseased glomeruli overwhelms the capacity of normal proximal tubular cells to reabsorb these proteins, injures the proximal tubule cells and results in tubulointerstitial damage and progressive CKD (Makhammajanov et al., 2024). In Lowe syndrome, tubular reabsorption is already abnormal due to disrupted endocytosis in the diseased proximal tubule, so albuminuria is a marker for tubular injury and an unreliable marker for glomerular injury and progressive CKD.

Central hypotonia occurs in infancy (Bokenkamp and Ludwig, 2016) and improves with age. Older patients have persistent elevation of serum muscle-related enzymes (creatine kinase, lactate dehydrogenase, aspartate aminotransferase). Muscle biopsies in 3 Lowe syndrome patients and one Dent 2 patient showed primary myopathy (Park et al., 2024) suggesting direct muscle injury in at least some of the patients with *OCRL* variants. Persistent decreased truncal motor tone increases the risk of developing scoliosis in these patients (Lewis et al., 2019). Affected teenagers and older adults may have joint swelling, arthritis, tenosynovitis and subcutaneous benign fibromas, often on the hands and feet and especially in areas of repeated trauma as from repetitive hand-biting (Lewis et al., 2019).

Bone disease may be related to phosphaturia with hypophosphatemia, decreased production of 1,25-dihydroxyvitamin D in the proximal tubule and chronic acidosis with the classic appearance of rickets on bone radiographs (Lewis et al., 2019). However, some boys with well-corrected Fanconi syndrome, normal serum 1,25-dihydroxyvitamin D levels and no radiographic findings of rickets have repeated pathologic bone fractures with poor healing suggesting a primary bone disorder. Inactivity, muscle hypotonia and immobilization in wheelchairs also may contribute to bone disease. Many patients have dental problems, including cavities (42%), misaligned teeth (67%) and mouth cysts (30%) (Lowenstein et al., 2024). Behavioral issues interfere with dental hygiene, frequency of examinations and procedures without general anesthesia making it difficult to know how much of the dental problems are due to the *OCRL* gene variant or to poor or unavailable dental care. About one third of Lowe syndrome boys have undescended testicles (cryptorchidism) (Lewis et al., 2019). Superficial epidermal cysts or eruptive vellus hair cysts may occur on the skin, especially the scalp, lower back and buttocks (Lewis et al., 2019; Bokenkamp and Ludwig, 2016; Goodman et al., 2023). Hidradenitis suppurativa, characterized by chronic deep skin nodules that abscess and develop fistulae and sinus tracts with scarring, occurs in patients with Lowe syndrome as well as Dent 2 disease (Lee J. H. et al., 2023). Little is known of the pathogenesis of these less common phenotypes in Lowe syndrome.

Thrombocyte dysfunction with prolonged or delayed bleeding after surgery, such as extraction of cataracts, occurs in Lowe syndrome (Lewis et al., 2019; Bokenkamp and Ludwig, 2016). Mild thrombocytopenia occurs in about 20% (Recker et al., 2015). In one study of 15 patients, about half had abnormal platelet function assays (Egot et al., 2021). Ocrl1 is highly expressed in thrombocytes and is needed for thrombocyte spreading and cytoskeletal shape rearrangements during primary clot formation (Egot et al., 2021; Bura et al., 2023). Ocrl1-deficient thrombocytes have defective actomyosin cytoskeleton reorganization for clot formation and retraction, impaired spreading on fibrinogen and formation of fewer proplatelets from downregulated megakaryocytes. These deficiencies contribute to the mild bleeding problems and thrombocytopenia seen in Lowe syndrome patients.

## Cellular phenotype variation

**FIGURE 1:**
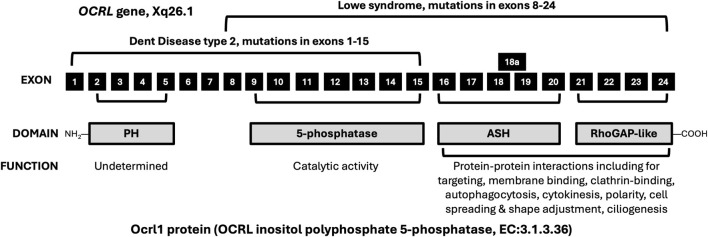
Schematic map of OCRL gene exons (black boxes not drawn to scale) that encode domains of the Ocrl1 protein (grey boxes) with functions of the Ocrl1 domains. Most mutations cluster in exons 8–24 for Lowe syndrome and exons 1–15 for Dent 2. Overlap with exons 8–15 may account for some of the overlap in clinical symptoms between the two diseases, although patients with *OCRL* mutation in the same location may have completely different clinical phenotypes (Zaniew et al., 2018; Hichri et al., 2011; Du et al., 2024; Ando et al., 2024; Akhtar et al., 2022). Function of the PH domain of the Ocrl1 protein, encoded by exons 2-5, is yet to be determined (De Sa et al., 2025). The PH domain contains a clathrin box motif, but it is not functional in the normally folded protein. The 5-phosphatase domain, encoded by exons 9–15, catalyzes dephosphorylation at the 5-position of the inositol ring in phosphoinositide lipids, especially phosphatidylinositol 4,5-bisphosphate. The ASH domain, encoded by exons 16–20, binds to Rab GTPases to guide proper targeting of Ocrl1 within cells. Alternative splicing of exon 18a between exons 18 and 19 in the *OCRL* gene leads to an isoform expressed primarily in the brain. The RhoGAP-like domain, encoded by exons 21–24, folds together with the ASH domain to form a functional unit that is essential for protein-protein interactions for signaling and trafficking in cells. NH2; N-terminal end of Ocrl1; PH, pleckstrin homology; ASH, ASPM-SPD2-Hydrin; RhoGAP, Rho GTPase activating; COOH; C-terminal end of Ocrl1.

Ocrl1 is a complex protein of 110 kDa with four domains (Figure 1): an N-terminal pleckstrin homology (PH) domain encoded by *OCRL* gene exons 2-5, a 5-phosphatase domain encoded by exons 9–15, an ASPM-SPD2-Hydrin (ASH) domain encoded by exons 16–20, and a Rho GTPase activating (RhoGAP)-like domain encoded by exons 21–24 (De Matteis et al., 2017; Ramadesikan et al., 2021; De Sa et al., 2025). Alternative splicing of an extra exon 18a between exons 18 and 19 in the *OCRL* gene leads to an isoform expressed primarily in the brain (Bokenkamp and Ludwig, 2016). PH domain function is unclear (De Sa et al., 2025). Even though the PH domain contains a clathrin box motif, it is not functional for clathrin binding in the normally folded protein. Clathrin binding, like that in early endosomes for LMW protein reabsorption in the proximal tubule, is mediated by the ASH-RhoGap-like domains. The 5-phosphatase domain catalyzes enzymatic dephosphorylation at the 5-position of the inositol ring in phosphoinositide lipids, especially phosphatidylinositol 4,5-bisphosphate. The ASH domain binds to Rab GTPases to guide proper targeting of proteins within cells. The RhoGAP-like domain does not activate Rho family GTPAse but forms a functional unit with the ASH domain at the C-terminal end of the protein. The ASH-RhoGAP-like domain is essential for protein-protein interactions for signaling and trafficking, including vesicle trafficking, autophagocytosis, cytokinesis, cell spreading, actin cytoskeletal remodeling and ciliogenesis. Ocrl1 is found in many tissues and in multiple subcellular locations, including plasma membrane, clathrin-coated vesicles, early endosomes, stressed lysosomes and Golgi apparatus. As expected from the complexity of Ocrl1 functions and expression in multiple tissues and subcellular locations, loss of function of Ocrl1 in Lowe syndrome results in heterogenous cell phenotypes (De Matteis et al., 2017; Ramadesikan et al., 2021; Lee J. J. et al., 2023). Cell phenotypes depend on the expression of variant Ocrl1 in the cell, including stable inactive Ocrl1 conformations (abnormal folding), misdirected subcellular locations, shortened or fewer primary cilia, defective membrane remodeling for cell spreading/cytokinesis/endocytosis, impaired actin cytoskeleton readjustments, impaired trafficking of stressed lysosomes and Golgi apparatus fragmentation.

Cell phenotypes are being studied in multiple laboratory models, including human embryonic kidney 293 T epithelial and proximal tubule HK2 cells (Ramadesikan et al., 2021; Lee J. J. et al., 2023), proximal tubular cells from transgenic mice (Berquez et al., 2020), neuronal cells induced from Lowe syndrome patient-derived pluripotent stem cells (Akhtar et al., 2022; Barnes et al., 2018), patient-derived platelets (Egot et al., 2021), Ocrl1-inhibited human thrombocytes (Bura et al., 2023) and transgenic zebrafish (Lowe, 2025). Studies in these model systems are leading to new insights about normal Ocrl1 functions/interactions, changes in the cellular milieu induced by *OCRL* variants and the potential contribution of cell phenotype to Lowe syndrome clinical phenotypes.

## Genotype/phenotype correlation

Even though Lowe syndrome is a monogenic disease, correlation of genotype with phenotype has proven to be complex. Additionally, two diseases, both Lowe syndrome and Dent 2, result from mutations in the same *OCRL* gene, and some patients even have mutations at the same gene site but different clinical symptoms (Zaniew et al., 2018; Hichri et al., 2011; Du et al., 2024; Ando et al., 2024; Akhtar et al., 2022). Of the over 300 disease-causing mutations of the OCRL gene, just over 200 are associated with Lowe syndrome and more than 50 are associated with Dent 2 (HGMD, 2021). Most are missense or nonsense variants (49%) followed by splicing (11%), small deletion (20%), small insertion (10%) or gross deletion (6%) variants. In Lowe syndrome, missense variants are predominantly localized to the 5-phosphatase domain (exons 9-15), and truncating mutations map exclusively to exon 8 (De Matteis et al., 2017). Lowe syndrome variants mostly occur in exons 8–24 with exon 15 being the most affected (Du et al., 2024) while those for Dent 2 tend to cluster in exons 1–15 (Figure 1). Dent 2 shares many of the same clinical features of Lowe syndrome (Table 1) but in a milder form, including LMWP, hypercalciuria, kidney calcinosis/stones, short stature (just <2 height SD) and extra-kidney involvement of muscle (elevated serum muscle-related enzymes), nervous system (cognitive/behavioral issues) or eye (mild cataracts but no congenital cataracts) but rarely all three systems (Table 1) (Gianesello et al., 2021). Even though mutations for Dent 2 cluster in exons 1–15, they are found in all 24 exons (Zaniew et al., 2018; Gianesello et al., 2021). Reports of the same mutation causing either Dent 2 or Lowe syndrome with different severity underscore the difficulty in using the *OCRL* genotype to predict clinical phenotype or severity (Hichri et al., 2011). Recent studies of *OCRL* variant transcripts (Sakakibara et al., 2022) and splicing assays in a minigene system (Rossanti et al., 2025) suggest that differences in Ocrl1 isoform expression leading to truncation or aberrant splicing help account for some of the genotype/phenotype variation between Dent 2 and Lowe syndrome. For example, truncating mutations in exons prior to exon 8, which contains the Ocrl1 translation initiation start site, leads to preservation of >50% enzyme activity and the milder phenotype of Dent 2 while mutations after exon 8 lead to <20% enzyme activity and the more severe phenotype of Lowe syndrome (Sakakibara et al., 2022).

Zaniew et al. (2018) studied 106 boys <19 years old with either Lowe syndrome or Dent 2 (Table 1) and found no difference in association of kidney outcomes with phosphatase domain variants compared to RhoGap-like domain variants. Ando et al. (2024) confirmed this finding in an older cohort (2-45 years old; 17 older than 16 years old) of 54 patients (51 families). Some significant trends for Dent 2 were noted by Gianesello et al. (2021); for example, ocular symptoms were more frequently present when mutations were in the ASH domain compared to the PH and 5-phosphatase domains (p < 0.01) and neurological symptoms were more frequent with mutations in the 5-phosphatase compared to the PH domain (p < 0.01). Zhang et al. (2022) found that truncating lesions that shorten Ocrl1 due to nonsense, splicing and incomplete insertion/deletion frameshift mutations were significantly more common in Lowe syndrome (34/48 patients; 71%) compared to Dent 2 (11/35; 31%), whereas non-truncating lesions that alter Ocrl1 function without changing its length and are due to missense or small in-frame insertion/deletion were more common in Dent 2 (24/35; 71%). They also found that most mutations in Lowe syndrome are located in exons 13-23, and the majority in Dent 2, in exons 2-12. These results suggest that different sets of mutations underlie the two diseases and that mutations in exons 10-15 may cause either disease. Why the same mutation may clinically manifest as Lowe syndrome or Dent 2 with different severity has yet to be determined.

Correlation of genotype with cell phenotype is also complex and varies by tissue. The most severely affected cell phenotypes tend to be in the most metabolically active tissues like kidney, brain and eye. Studies of kidney and neuronal cells are ongoing and providing new insights into genotype/phenotype correlation (De Matteis et al., 2017; Ramadesikan et al., 2021; Akhtar et al., 2022; Lee J. J. et al., 2023). Song et al. (2017) reviewed 30 Lowe syndrome patients with congenital cataracts and glaucoma and correlated these eye abnormalities with clustering of missense and deletion *OCRL* gene mutations in the 5-phosphatase and RhoGap-like domains. In thrombocytes, Ocrl1 expression and function primarily affects thrombocyte shape for clot formation (Egot et al., 2021; Bura et al., 2023), so cell phenotype in thrombocytes may be less complex to study. Genotype/phenotype correlations have yet to be evaluated in thrombocyte dysfunction.

## Discussion

The “what” in what clinical variations occur in Lowe syndrome is well known and described in the Clinical Phenotype Variation section. The “how” of how mutations in the *OCRL* gene lead to different clinical and cellular phenotypes is still elusive. Even though Lowe syndrome is a monogenic disease, no singular mechanism explains the clinical phenotype, likely reflecting the presence of the *OCRL* gene product Ocrl1 in many tissues with variable expression, subcellular location and complex functions. The same *OCRL* gene variant is known to cause clinical features of Lowe syndrome in one patient and of Dent 2 in another and to manifest different features or different severity of features between families and even within affected boys of the same family (Zaniew et al., 2018; Hichri et al., 2011; Du et al., 2024; Ando et al., 2024; Akhtar et al., 2022). Potential factors which may contribute to the complexity of correlating genotype with phenotype include: a) compensatory and variable correction of Ocrl1 function by corrector genes like *INPP5B,* which encodes for inositol polyphosphate-5-phosphatase-5B that shares significant sequence identity, domain organization and function with Ocrl1; b) presence of unrelated enhancer genes that are inherited differently within families; c) need for full 5-phosphatase activity for catalytic as well as non-catalytic functions of Ocrl1; d) partial expression of normal function by shortened or aberrantly spliced Ocrl1 variants as in Dent 2 (Sakakibara et al., 2022; Rossanti et al., 2025); and/or e) contributions from the extracellular matrix in different tissues (De Matteis et al., 2017; Ramadesikan et al., 2021; De Sa et al., 2025; Akhtar et al., 2022).

Continued study of specific Ocrl1 functions and cell phenotypes may lead to better correlation of genotype to cell phenotype in this rare disease and to identification of subsets of patients with the same abnormality that might be amenable to therapeutic intervention, such as with repurposed drugs (De Matteis et al., 2017; Berquez et al., 2020).
